# Easing Power Consumption of Wearable Activity Monitoring with Change Point Detection

**DOI:** 10.3390/s20010310

**Published:** 2020-01-06

**Authors:** Cristian Culman, Samaneh Aminikhanghahi, Diane J. Cook

**Affiliations:** School of Electrical Engineering and Computer Science, Washington State University, Pullman, WA 99164-2752, USA; cristian.culman@wsu.edu (C.C.); s.aminikhanghahi@wsu.edu (S.A.)

**Keywords:** time series analysis, machine learning, mobile computing, statistical methods, energy reduction

## Abstract

Continuous monitoring of complex activities is valuable for understanding human behavior and providing activity-aware services. At the same time, recognizing these activities requires both movement and location information that can quickly drain batteries on wearable devices. In this paper, we introduce Change Point-based Activity Monitoring (CPAM), an energy-efficient strategy for recognizing and monitoring a range of simple and complex activities in real time. CPAM employs unsupervised change point detection to detect likely activity transition times. By adapting the sampling rate at each change point, CPAM reduces energy consumption by 74.64% while retaining the activity recognition performance of continuous sampling. We validate our approach using smartwatch data collected and labeled by 66 subjects. Results indicate that change point detection techniques can be effective for reducing the energy footprint of sensor-based mobile applications and that automated activity labels can be used to estimate sensor values between sampling periods.

## 1. Introduction

Observing, recognizing, and analyzing human activities form a foundation for scientific fields such as anthropology, archeology, sociology, and psychology. With the maturing of wearable sensors and computers, a person’s activities can now be monitored around the clock via mobile sensors. What is more, given the 127 million smartwatches that were sold last year alone, the volume of already-collected activity is unprecedented. Researchers can analyze this data to validate theories of human behavior and practitioners can gain insights that allow them to provide personalized recommendations and treatment plans. The impacts of this “activity wave” are profound. The earliest wearable fitness trackers debuted over a decade ago. Building on their foundation, researchers have applied these mobile technologies to cognitive and physical health monitoring [1,2,3,4], activity-aware recommendations [5], sports evaluation and training [6], lifelogging [7], and behavior intervention [8].

To provide quality activity-aware services [9,10], mobile devices must be worn nonstop and must be continuously collecting data without interruption. At the same time, frequent sensing and user localization can quickly drain a smartwatch battery. In this paper, we introduce Change Point-based Activity Monitoring (CPAM), an algorithm that performs continual monitoring and recognition of activities of daily living while using change point detection and change point-adaptive sampling to reduce energy consumption. Adopting the CPAM strategy results in a saving of 74.64% in energy consumption, extending battery life and thus the usefulness of activity-aware applications.

Energy consumption is a known obstacle to wearable computing in general and to activity monitoring in particular [11,12,13,14,15]. For complex activities, however, recognition and monitoring may require an even greater energy footprint. While many approaches use movement sensors to recognize atomic movement-based activities (e.g., sit, stand, walk, climb, run, lie down), additional information such as location is needed to learn activities of daily living that often contain combinations of basic movements (e.g., cook, watch television, work). Our long-term goal is to recognize such complex activities, in real time as people perform them. To achieve this goal, we must create new approaches to sensing that reduce energy consumption and save battery life.

We hypothesize that mobile energy consumption can be dramatically reduced while recognizing activities of daily living in real time by recognizing natural changes in state (e.g., activity transitions) and adapting sampling rates to these changes. Here, we describe the CPAM algorithm that performs energy-efficient activity recognition. CPAM collects sensor and location data, detects activity changes, adjusts the sampling rate correspondingly, and recognizes activities of daily living in real time. We evaluate our method for wearable data collected from 66 users, labeling nine basic and instrumental activities of daily living. We also investigate an enhancement to CPAM that uses activity labels to estimate sensor values between sampling periods as a strategy to further reduce sampling rates while maintaining the ability to accurately detect and recognize critical activities.

## 2. Related Work

Because the need for continuous sensing is juxtaposed with the need for long battery life, researchers have presented numerous options for reducing energy consumption while performing activity recognition and context-aware mobile computing. One such paradigm is compressive sensing [16]. Compressive sensing maintains that a signal does not have to be sensed equally at all times to achieve a standard of information quality. Instead, when the signal is sparse, the sampling rate can be reduced and signals can be compressed. Naturally, the resulting information quality depends on the capability of the signal receiver to reconstruct the original information. Mobile sensors generate much redundant data that spark unnecessary computation, storage, and transmission [17]. Therefore, researchers have explored this methodology to improve mobile power efficiency for applications in biomedical computing. For example, Mamaghanian et al. [10] compress ECG data before transmission, extending mote lifetime by 37.1%. Elgendi et al. [18] achieve a compression ratio of 6 for ECG data, while retaining 99.56% reconstruction accuracy.

Compressive sensing has been investigated specifically for mobile activity monitoring by researchers such as Akimura et al. [11], who reduce power consumption by 16% while maintaining a recognition accuracy of over 70% for scripted the motion-based activities stay, walk, jog, skip, climb up stairs, and descend down stairs. Similarly, Jansi and Amutha maintain f-score, specificity, and precision as well as accuracy for recognition of eight movement-based scripted activities using compressive sensing with a sparse-based classifier [12]. Hui et al. found that they could directly use the compressed information to recognize six activities with an accuracy of 89.86% when combining compressive sensing with strategic placement of the mobile device on the body, and Braojos et al. [19] quantify the precise relationship between wearable transmission volume and activity recognition sensitivity.

The flipside of reducing the mobile energy footprint is making needed power available through energy harvesting. Human motion not only reflects current behavior, but it can also be converted into power. Some researchers such as Khalifa [13] and Lan et al. [20] transform kinetic energy into mobile power. At the same time, they directly analyze the kinetic energy harvesting patterns to detect and analyze human activity. In our work, we do not compress and reconstruct the signal, nor do we harvest energy. Instead, we control how often the signal is sampled. However, our adaptive sampling could be combined with compressive sensing and energy harvesting to potentially yield even greater resource savings.

In the same way that distributed computing lightens computational loads for each node, so distributed sensing lessens the need for energy-consuming sensing for each mobile device. Kwak et al. [21] share sensed locations between nearby mobile devices, while other groups such as Guo et al. [22] offload sensor processing to the cloud. To intelligently and fairly assign sensing efforts among available nodes, Sheng et al. [23] create a separate controller that makes these decisions. Based on simulated scenarios, they identify the minimum number of readings that sensors must provide for successful applications. Our work contrasts from distributed computing approaches in that we do not rely on multiple devices or external servers. This allows each device to operate as an independent entity.

One powerful role for mobile devices is to monitor and label user activities. This role places heavy demands on mobile devices for continuous sensing. Not only is long battery life essential for continual monitoring, but Alshurafa et al. [24] found that it is also essential for maintaining intervention adherence. Battery drainage causes interruptions to an intervention plan and thus discourage users from participating. Uninterrupted intervention thus represents an additional motivation for adaptive sampling. Alshurafa et al. extended battery life by downsampling when the accelerometer indicated the user was not in motion. This strategy improved intervention compliance by 53%. Gordon [14] adopted a similar approach but also predicted future activities. Inferring likely upcoming behavior allows sample rates to be adjusted in anticipation of the next activities. Yan et al. [25] specifically selected sampling frequencies based on a formalized trade-off between activity classification and accuracy. Pagan et al. [15] and Fallahzadeh et al. [26] incorporate insights about activity-specific sensing granularity as well as compressive sensing to enhance this trade-off.

Other researchers found that low-power activity recognition may rely on more effective use of the sensing device. Grutzmacher et al. [27] and Elsts et al. [28] relegate the feature extraction work to the device rather than the server, which lowers the overall energy consumption because of a decreased need for data transmission. Bhat et al. [29] found that they could achieve activity recognition accuracy as high as 97.7% even with a low-power IoT device, and Braojos et al. [19] achieved up to 97.2% accuracy with low-power wearable nodes.

Another noticeable impact on power consumption is the choice of software architecture. Berrocal et al. [30] demonstrated how dramatically choices of software architecture varied battery and data traffic consumption. In particular, server-centric architectures become more efficient as interactions with external entities increase, while mobile-centric architectures may be preferable if the shared data require frequent updates.

All of these approaches to power-sensitive activity monitoring have been directed toward sensing of activities that are scripted, evaluated in controlled settings, and are distinguishable based on a single type of body movement. In our work, the goal is to monitor and recognize complex activities of daily living with wearable devices from streaming data as activities are performed in everyday, realistic settings. Not all activities of daily living contain a single atomic type of movement. When activities are considered that contain combinations of movements and locations, activity transition detection will play a key role because transitions dictate when sampling rates should be increased and decreased. French et al. [31] also offer a strategy that is based on this philosophy. In particular, they sample sensors only at activity transitions. In their experiments with 94 h of collected continuous data for 4 users, they were able to accurately label 11 activities with only 10%–20% of the available samples using this technique. However, their work relied on manual identification of activity transitions. We replace this step with automatic transition labeling via change point detection.

## 3. CPAM

**Figure 1:**
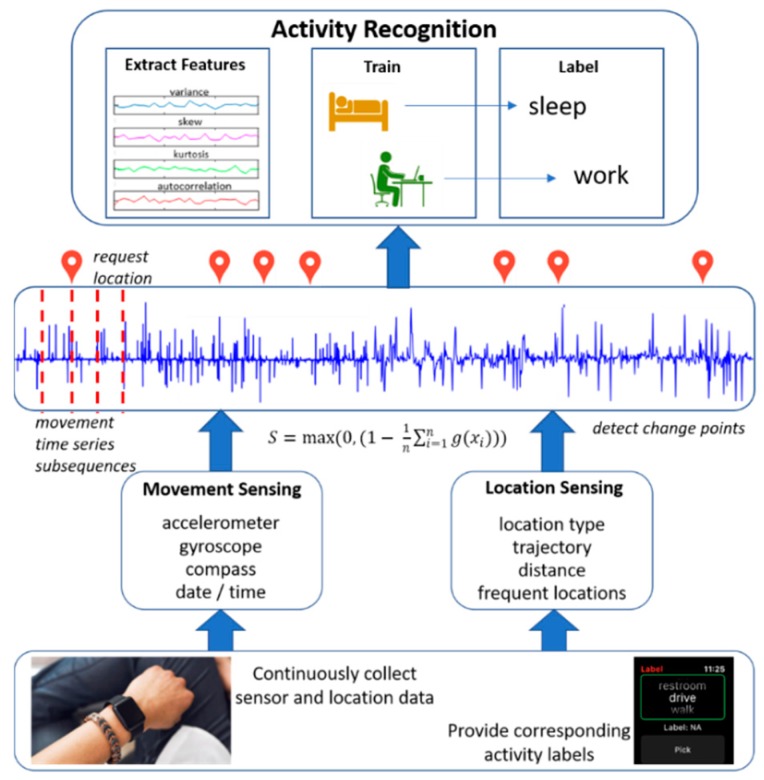
Overview of the Change Point-based Activity Monitoring (CPAM) energy-conserving activity recognition algorithm. Data are continuously collected and are either labeled by users in real time to train the model or labeled by the model once it is trained. Movement and location data are analyzed to find change points and sampling rates are adjusted accordingly. The resulting data are fed to a supervised learner to build activity models.

We hypothesize that sampling sensors at times indicated by change points, or changes in the process state, can reduce energy consumption while maintaining a high quality of service for mobile applications. We validate this hypothesis for an activity recognition smartwatch app called CPAM (Change Point-based Activity Monitoring). Figure 1 provides an overview of CPAM. As shown, smartwatch users continuously collect sensor data during their normal daily routines, using the app to provide labels for their current activities. The collected data are stored together with user-provided labels on the watch. Upon user request, the data can also be securely transmitted to a password-protected database on a remote server.

Two types of sensor data are collected for activity monitoring. Movement sensors generate acceleration, gyroscope, compass, and heart rate data. We postulate that location data are critical for recognizing complex activities and these are separately collected and stored. However, obtaining location data consumes a much greater amount of energy. CPAM acts as a closed-loop system to obtain essential data at rates that are sensitive to the current recognition needs. As the data are collected, CPAM analyzes data subsequences to find changes in state, or change points. When a change point is detected, the sampling rate is increased to support activity recognition. The sampling rate is then decreased until the next change point, based on the assumption that the current activity persists until the change point. Finally, sampled data are provided together with activity labels to train a machine learning classifier. This classifier learns activity models and can use them to label new data with the corresponding activity categories in real time.

### 3.1. Monitoring Complex Activities

While CPAM can provide activity-aware energy reduction for many mobile applications, here, we focus on an activity recognition application. Human activity recognition is a popular research topic [32,33,34,35] and forms a critical component of technologies for health monitoring, intervention, and activity-aware service provisioning [36,37,38]. Additionally, activity recognition provides a vehicle for us to validate our change point detection methods by comparing detected activities with known activity transitions.

**Figure 2:**
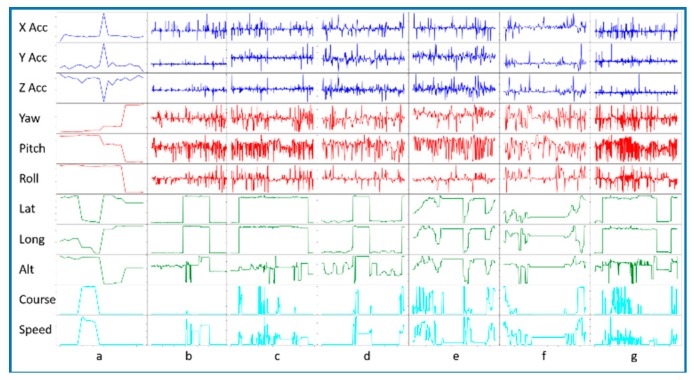
Graph displaying sensor values for a single user’s monitored activities. Sensed values are (x, y, z) acceleration, yaw, pitch, roll, latitude, longitude, altitude, course, and speed. The corresponding activity categories are a = sleep, b = work, c = eat, d = exercise, e = travel, f = hygiene, and g = hobby.

**Figure 3:**
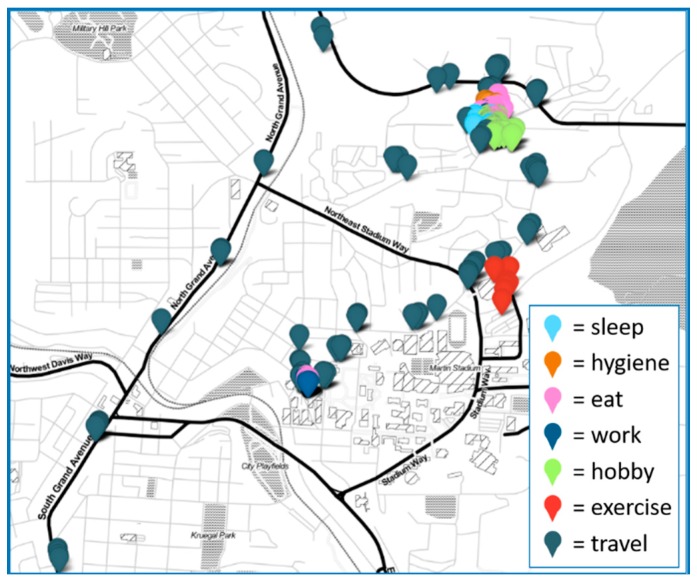
Map of locations visited over the course of a single day for one user with corresponding activity labels.

In this work, we propose an algorithm to recognize activities of daily living in real time. Some activities of daily living consist of a single type of position and movement (e.g., sleep). In contrast, other activities, what we refer to as complex activities, may combine any number of movement types (e.g., errands may combine sitting, standing, and walking). Additionally, some activities of daily living cannot easily be distinguished based on movement alone. For example, watching television and listening to a lecture utilize very similar movements. These activities need additional information including date, time, and location to be recognized. Figure 2 and Figure 3 show the diversity of information that is provided by the different types of CPAM sensor readings, including both movement and location. This diversity of information is essential to distinguish the activity categories.

Modeling, recognizing, and monitoring complex activities is essential for several reasons. First, health professionals often use a person’s ability (or inability) to perform activities of daily living (ADLs) as a measurement of their health status. These include basic ADLs such as personal hygiene, moving independently, and self-feeding as well as instrumental ADLs (iADLs) such as cooking, shopping, traveling, handling finances, and performing household chores [39]. Assessing a person’s functional health is critical not only for monitoring changes in health state but also for determining the impact of interventions [40].

Second, complex activities form a vocabulary that is typically used to express human behavior. As an example, the American Time Use Survey (ATUS) [41] catalogs the percentage time that people spend on “typical” activities. Here, activity categories include eating, leisure, sports, sleeping, working, household activities, and caring for others.

**Figure 4:**
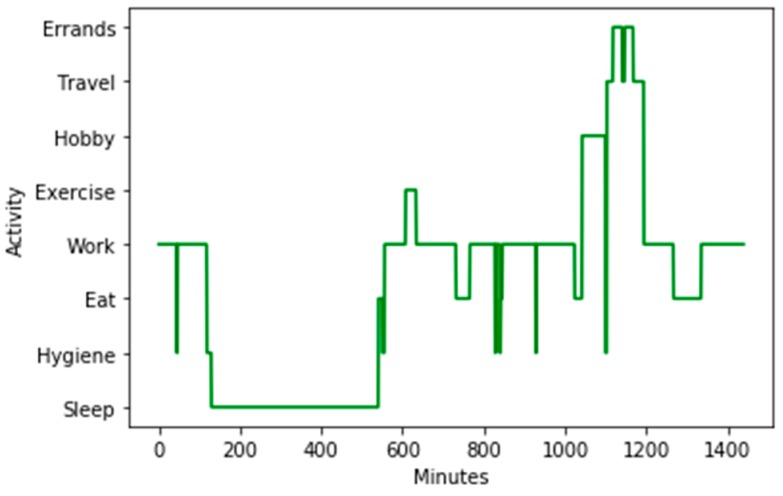
A one-day sequence of activities and corresponding activity durations for one of the users. Time on the x axis starts and ends at midnight.

We collected data for a collection of activities that encompass the ADL, iADL, and ATUS categories. To ensure that we maintained a consistency of label interpretations and collect a sufficient number of instances for each category, we group some of the specific activities. These groupings are listed in Table 1 together with the corresponding set of labels provided by our users. To visualize routine behavior based on these activity categories, Figure 4 shows a sample one-day activity sequence for one of our users.

### 3.2. Real-Time Activity Recognition

Activity recognition maps sensor data to corresponding activity labels using supervised machine learning. Input to an activity learner is a sequence of sensor events. A sensor event takes the form *e_t_ = <t*, *r*_1_, *r_d_>*, where *t* denotes the date and time of the set of sensor readings and *r*_1_ through *r_d_* indicate values returned from the collection of *d* sensors at time *t*.

Many activity recognition approaches extract features corresponding to an entire pre-segmented, scripted activity and map the feature vector onto a corresponding activity label. In contrast, CPAM maps continuously-collected smartwatch data onto activity labels in real time. To accommodate this difference in approach, CPAM moves a sliding window over the data. For this paper, the window size, *w*, is set to 5 s motivated by experiments reported from our group and others [42,43]. Features are extracted from a window and the supervised learning algorithm maps this feature vector onto an activity label, <*f_statistical_, f_relational_, f_temporal_, f_navigational_, f_personal_*, *f_positional_>→A*. Table 2 summarizes CPAM’s sampled sensors, the extracted features, and the category of sensor data for which the features are derived. Activity categories that were reported by a majority of the users are included in the study as listed in the table.

The app samples 3D acceleration and rotation readings together with course, speed, device orientation, user heart rate, and the date and time of the sample. Additionally, location services are used to collect latitude, longitude, and altitude readings. For each data window, or time-ordered sequence of sensor readings, features are extracted. Statistical features are calculated independently for each sensor based on the readings within the window. Relational features combine two or more sensors. For example, correlations are calculated between the multiple acceleration axes. Rotational and locational correlations are calculated in a similar fashion. The navigational features consider the number of times a user’s course changes within the window (heading change rate), the number of stops and starts within the window (stop rate), the trajectory vector from window beginning to ending, and the distance that was traveled during that time.

While we contend that location information is valuable for many mobile services including activity recognition, reasoning about specific <latitude, longitude, altitude> locations does not allow learned models to generalize over multiple users. Furthermore, a model built on this information could jeopardize the privacy of the users on which it was built. Instead of including specific locations in the model, we extract generalizable location features. For each user, we identify the top 6 overall frequent locations and most-frequent locations by time of day (midnight to 06:00, 06:00 to noon, noon to 18:00, 18:00 to midnight) using k-means clustering with a Euclidean distance metric. These are created based on an initial sample of data for each user. For new data, cluster memberships are identified, and the cluster IDs are added to the feature vectors. We also calculate the geographic center of all locations the user visits and incorporate a feature that represents the x distance, y distance, and Euclidean distance of a given location from the user center. These distances are normalized based on the bounding box around the user’s frequent locations.

Finally, we extract a feature that represents the location type. Given a sampled location, we use the Nominatum open street map to generate a corresponding address and the location type. We group these into the location categories home, restaurant, road, store, work, attraction, service, and other, then use one hot encoding to include location type in the feature vector. Because accessing the open street map requires communication that further drains the battery, we learned a separate model that maps the non-location features from Table 2 onto a corresponding location type. The model achieved 98.1% classification accuracy for 3-fold cross validation on 20,000 reverse geocoded locations previously collected from individuals living in the same geographical regions as the participants in the CPAM study. After validating the model, we trained it on all 20,000 locations and employed the learned model to generate location types on the CPAM smartwatch app.

### 3.3. App Design

**Figure 5:**
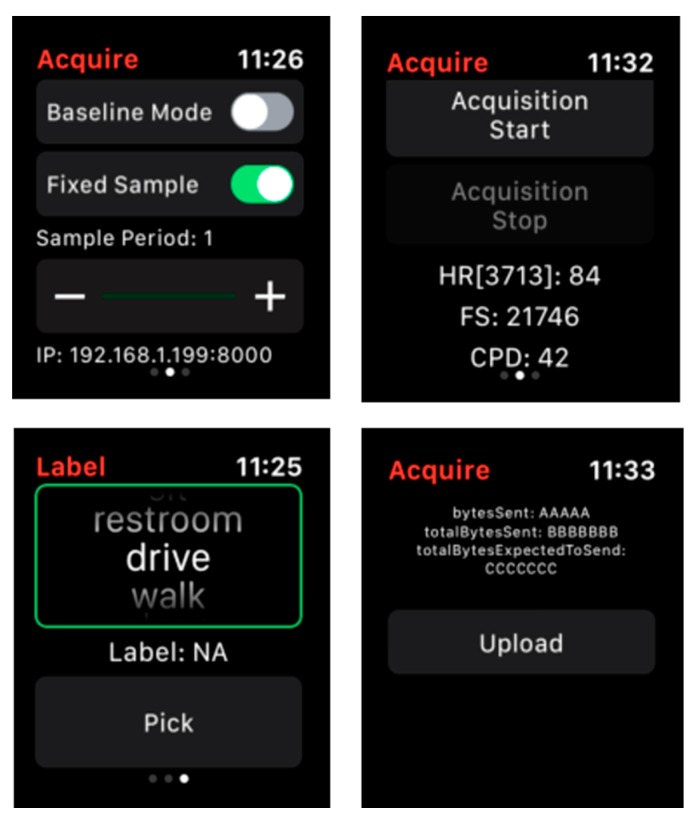
Screenshots of the CPAM app. These functionalities allow the user to (**top left**) specify the sampling rate, (**top right**) start and stop data acquisition, (**bottom left**) provide a label for the current activity, and (**bottom right**) send collected and labeled data to a server.

CPAM is implemented in the Apple Watch 3. The app samples data at 100 Hz and provides an interface through which a user can label their current activity, start and stop data acquisition, and upload all collected data with activity labels to an offsite server. Figure 5 provides screenshots of these app functions. Models are updated on the watch periodically (currently once each day) and are similarly updated on the server to perform sample-wide data analysis and evaluation of activity recognition performance.

Activity recognition is performed on the watch using the CoreML libraries. Earlier experiments indicated that random forest with 100 trees performs well on activity recognition from wearable data [42] and we utilize this algorithm for CPAM. The collected features are generalizable, so we build a model that can be used for any existing or new user. Because the data are not uniformly balanced among the nine activity categories, training samples are given a weight that is inversely proportional to the size of their activity class. For future versions of CPAM with more activity categories, sampling may need to be added to learn a sufficiently robust model for all of the activity classes.

### 3.4. SEP Change Point Detection

Change point detection refers to the process of finding points in time series data where the data-generating process changes. If data before time t reflects a different process state than data after time t, we can say that time t is a change point. Formally, given a time series stream of elements X = {x1,..., xi,...}, xi represents a d-dimensional feature vector arriving at time i. Each feature vector reflects a current state of the underlying process. Two consecutive distinct states appear on either side of a change point. Thus, the change point represents a transition between the corresponding states. In the case of activity-driven sensor data, the change point represents a transition between activity classes. Change point detection offers one method for segmenting time series data, by partitioning data between change points into separate, non-overlapping, varying-size time series segments. We hypothesize that activity transitions can be characterized as change points, and there is some evidence in the literature to support this claim [44,45].

**Figure 6:**
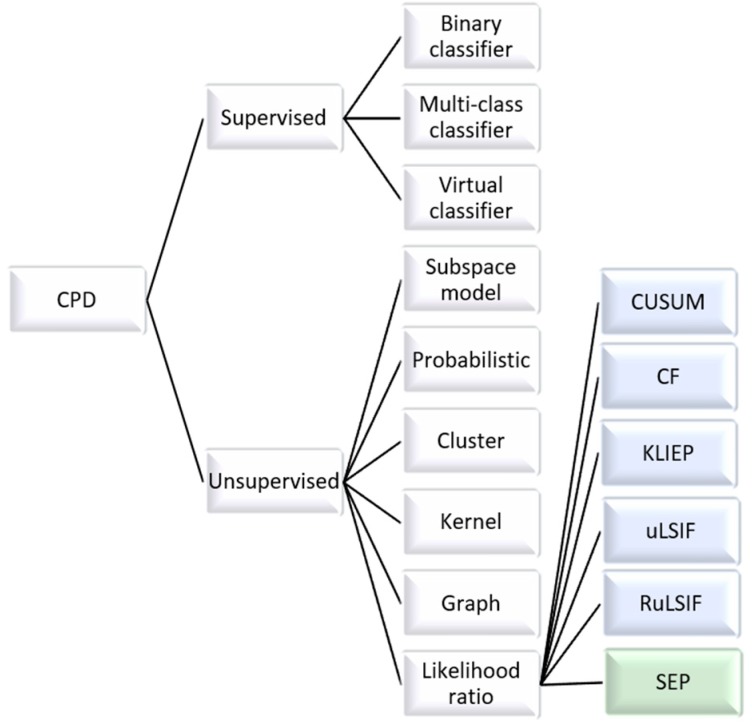
Recent approaches to change point detection.

While change point detection (CPD) is a thoroughly-investigated topic, some traditional approaches to change point detection, shown in Figure 6, are not appropriate for this problem. Supervised approaches are trained on sample change points [46]. They can be very effective, but they require training on a sufficient number and diversity of labeled examples, which makes them less useful for a variety of activity data. These training data may provide examples of change point versus non-change point sequences (for binary classification) or of transitions between specific process states for multi-class classification.

In contrast, unsupervised methods look for changes in data. These changes can be a quantitative distance between states as with subspace modeling [47], membership in different clusters [48,49], or a distance value generated by a kernel function or a graph [50]. Alternatively, the probability of a change point can be computed using Bayes’ theorem [51] or a Gaussian Process prediction [52].

One requirement of our proposed method is to detect change points from streaming data. While earlier methods perform batch processing, this constraint can be met by density ratio techniques. CUSUM [53] and CF [54] identify change points when the probability density of a data sequence before the point sufficiently differs from the data sequence after the point. KLIEP [55], uLSIF [56], and RuLSIF [57] improve the detection runtime by directly estimating the ratio of the probability densities. Recent research in activity segmentation parallels this change point research, including supervised learning of activity transitions [58,59,60,61,62], calculation of change point Gaussian probabilities [63], or application of a direct density ratio unsupervised method [45,64,65].

For our change point approach to mobile energy reduction we propose SEP, a SEParation distance strategy, because we showed it to be more sensitive to subtle changes in sensor time series data than other unsupervised methods and it is non-parametric [45,66]. Using SEP, time point *t* is considered a change point if the probability density function f created from the subsequences before and after *t* are different in terms of the density function parameters. For a random variable *X* defined on ℜ, function *μ_x_* calculates the probability density for subsets *B* of ℜ:

*μ_x_*(*B*) = *P*(*X*) (1)

Here, (ℜ,*B,μ_x_*) is a probability space and *P* represents the probability of *X* ∈ *B*. Assuming that two probability densities, *f_t−_*_1_(*x*) and *f_t_*(*x*), correspond to the two subsequences from time series *X* appearing immediately before and after time *t*, SEP uses a dissimilarity measure to quantify the difference between the probability densities. This measure, S*, can be used to determine if *t* represents a change point. Because SEP needs to compare probability densities before and after change points, there is a delay between change point detection and the current time. This delay corresponds to the length of the subsequences that are considered, *n*.

The separation distance *S* between time series subsequences is calculated using Equation (2).

(2) $$S^{*}=\max\left(1-\frac{f_{t-1}\left(x\right)}{f_{t}\left(x\right)}\right)$$

Instead of calculating each term in Equation (1), which is computationally costly, SEP estimates the probability density ratio using a Gaussian kernel function *g*(*x*), defined in Equation (3).

(3) $$g_{t}\left(x\right)=\frac{f_{t-1}\left(x\right)}{f_{t}\left(x\right)}=\sum_{i=1}^{n}\theta_{i}\prod_{j=1}^{n}K\left(x_{t}^{i},x_{t-1}^{i}\right)$$

The kernel function parameters are estimated by performing cross validation within the points *x_i_, .., x_i+n_* comprising the subsequence. The ratio is bounded below by 0 to avoid negative distance values. The output change point score, *S*, is defined in Equation (4).

(4) $$S=\max\left(0,\left(1-\frac{1}{n}\sum_{i=1}^{n}g\left(x_{i}\right)\right)\right)$$

Values of *S* are compared with a threshold α to identify change points. Thresholds vary by data type and are identified through experimentation with a data sample. Because state (activity) transitions can trigger several large change point scores in a row, SEP reports only local maxima as detected change points.

## 4. Experimental Results

We are interested in answering the following questions.

1. Can SEP accurately find change points in smartwatch sensor data that represent activity transitions?
2. Are location data essential for recognition of complex activities? To answer this question, we will compare activity recognition performance using only location data, using only movement (non-location) data, and using a combination of data sources.
3. How does CPAM compare with baseline methods for activity recognition performance?
4. How does CPAM compare with baseline methods for battery consumption?
5. Can CPD-based activity segmentation and activity recognition be used to infer location information for use with other context-aware applications?

We address each of these questions experimentally in the following sections.

### 4.1. Experimental Conditions

**Figure 7:**
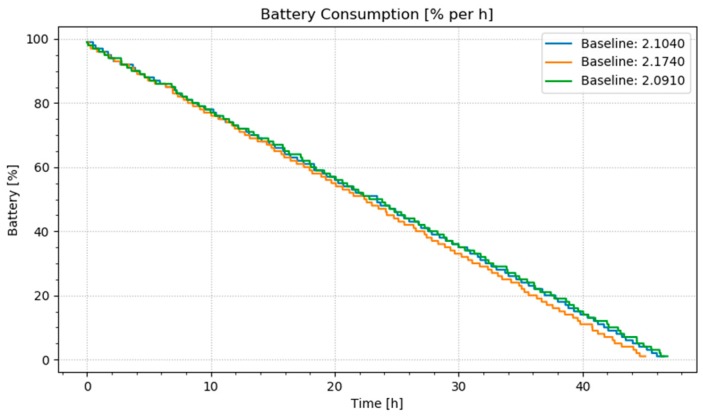
Baseline energy consumption on smartwatch over three runs from full charge until battery depletion.

For our experiments, we collected data from 66 volunteer young adult subjects. Subjects wore Apple watches with identical system settings on their non-dominant arm. No activities were scripted for these experiments—subjects performed their normal daily routines and used the smartwatch app interface to record activities in real time. No other apps were used on the watch during data collection. Figure 7 shows the baseline energy consumption of the watch when no apps, location services, heart rate/fitness tracking, or information-sharing features are enabled. As the graph shows, consumption is linear at approximately 2.104% of the total watch charge per hour.

Table 3 provides a breakdown of the collected data into the corresponding activity categories. Specifically, we list the number of sensor readings that were recorded for each category. We additionally record the number of activity occurrences for each category, where all sensor readings in a sequence that are labeled with the same activity are considered part of one activity occurrence. The total number of transitions is 46,229, computed as the total number of occurrences—1 (the last activity in the combined sequence).

**Figure 8:**
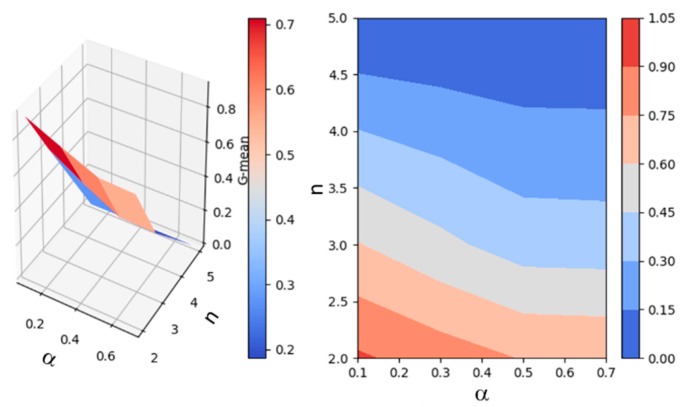
Selection of SEP algorithm parameters for smartwatch data based on a performance analysis for a sample of the first (*n* = 10,000) data points.

SEP algorithm parameters were selected based on a sensitivity analysis performed on a sample of the data. These parameters include the change point score threshold value as well as the length of subsequences to consider before and after each change point. As Figure 8 illustrates, a threshold value of α = 0.4 and a subsequence length of *n* = 2 were optimal for the data sample and were thus employed for the experiments.

### 4.2. Analysis of SEP for Smartwatch Data

We evaluated the performance of SEP change point detection on our activity-labeled sensor data using a g-mean score. This metric is a common performance measure for change point detection algorithms because of the extreme imbalance between change points and points that remain within the current state. G-mean computes the square root of the product of change point recognition sensitivity and specificity, where change points represent the positive class. Table 4 summarizes the performance of SEP on the sensor data. As the table shows, a majority of the actual change points are discovered, although some changes are also reported that are not due to actual transitions between activity classes.

As a baseline for comparison, we also computed performance for a baseline method that reports a change every five minutes (the length of the shortest observed activity). SEP performs significantly better (*p* < 0.05) than the baseline method.

### 4.3. Recognition Based on Movement and Location

Second, we consider the importance of movement information and location information for human activity recognition based on smartwatch data. To analyze the impact of these features, we perform activity recognition for the 66 subject smartwatch data using the nine activity classes listed in Table 2. For these experiments, we employ the activity recognition algorithm described in Section 3.2. Here, we compare performance using only “movement” data (acceleration, rotation, orientation, heart rate, date, time) with performance using all collected data (movement and location). Because the data are not uniformly distributed among the multiple activity classes, we report both recognition accuracy and macro f-score. F-score is computed separately for each activity class as (2 × Precision × Recall)/(Precision + Recall) and is averaged over all classes. All results are collected using 3-fold cross validation.

**Figure 9:**
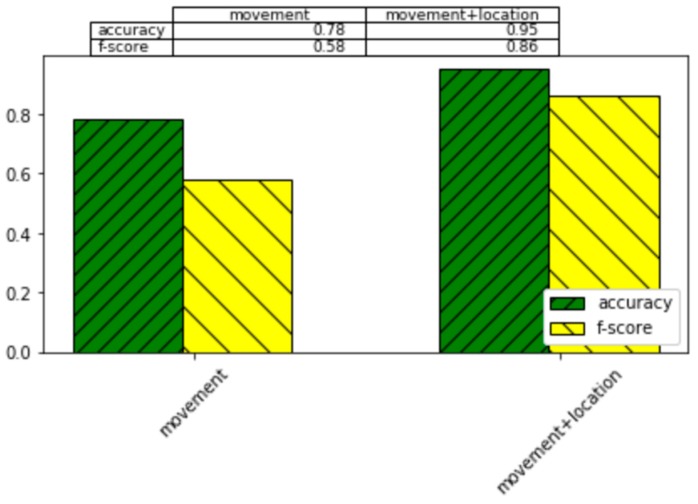
Activity recognition performance for 66 subjects based on 3-fold cross validation. Accuracy and macro f-score performance are reported for movement sensors, location sensors, and all sensors.

Experiment results are displayed in Figure 9. As the graph shows, recognition of activities of daily living benefits from sensing both movement and location. This finding is confirmed by both accuracy and f-score measures. Here, the difference in performance between movement-only sensors and movement sensors combined with location information is statistically significant (*p* < 0.05). Future work may reveal that the role of movement sensors is more impactful than location sensors for the recognition gestures and ambulation categories such as sit, stand, lie down, and run.

**Figure 10:**
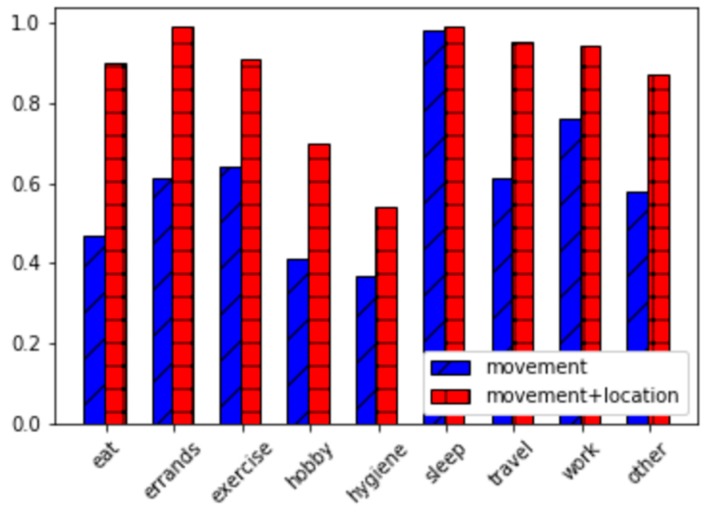
F-score activity recognition performance using only movement features and using movement combined with location features. Results are plotted for each activity category.

Next, we further analyze these two data sources by considering the f-score for each individual activity category using movement features and combining movement with location features. These results are plotted in Figure 10. These f-scores highlight which activities depend most heavily on location information. The activities that are most dramatically impacted (based on difference in f-scores) are eat, errands, travel, and hobby. The results are intuitive because these activities are easily distinguishable based on location type (e.g., restaurant, store, highway, movie theater) and movement alone may not be as distinct for the activity categories. In contrast, sleep is almost as easy to recognize with movement sensors alone as when all sensors are used. The type of movements and the body orientation are quite different than for other activity categories, so movement sensors alone are likely sufficient in this case.

### 4.4. Recognition Comparison with Baseline Energy-Reduction Methods

**Figure 11:**
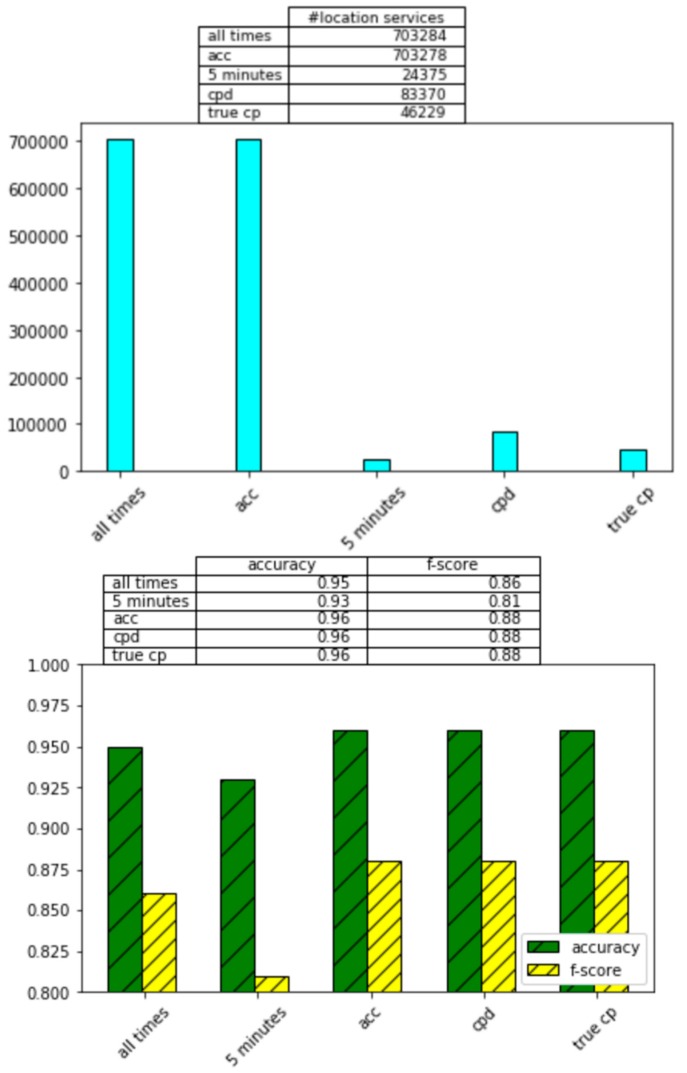
(**top**) Activity recognition accuracy for all times, acc, 5 min, CPD (CPAM), and true change point sampling strategies. The following accuracy and f-score differences are statistically significant. (*p* < 0.05): acc and all times, acc and 5 min, CPD and all times, CPD and 5 min, true cp and all times, true cp and 5 min. The changes in activity recognition performance between acc, CPD, and true cp are not statistically significant. (**bottom**) The number of location samples that are collected for the all times, 5 min, acc, CPD (CPAM), and true cp sampling strategies.

While it is apparent that including location information is important for recognition of activities of daily living, this information comes at a price of a dramatic increase in energy consumption. We hypothesize that CPAM can greatly reduce this energy consumption while retaining strong activity recognition and monitoring performance. In Figure 11, we observe the impact of the CPAM change point detection (CPD) method and two baseline methods on activity recognition performance.

The first baseline method (acc) samples location periodically rather than continuously. Rather than considering the data itself as an indication of when location is needed, the baseline method collects user location information every five minutes then turns it off until the next five-minute increment is reached. Sensing activity information every five minutes represents a strategy that has been previously used to monitor activity without draining the battery [42]. Additionally, the shortest monitored activity is approximately five minutes. Therefore, this selected sampling interval should be sensitive to even the quickest activity transitions.

The second baseline method (5 min) is based on movement, rather than time. Because a change in location implies that the user has moved, this baseline approach samples location whenever the sensed acceleration is a non-zero value. We also report performance for sampling location at ground truth change points (true change points, actual recorded transitions between activities). Finally, we record performance when location is sampled at every time interval (all times). As Figure 11 shows, CPAM performs comparably to the true change point method and superior to the baselines.

### 4.5. Energy Reduction

The activity recognition experiments in the previous sections highlight the tradeoff between number of location samples and reliability of models that are learned from the sample data. Our experiments record energy consumption as a function of percentage of battery that has been consumed. Given that the smartwatch battery capacity is 1.27 Wh and our experimental continuous observations for a single user over a two-week period, Table 5 summarizes the energy that is consumed by normal watch operations, by a single movement sample, and by a single location sample.

**Figure 12:**
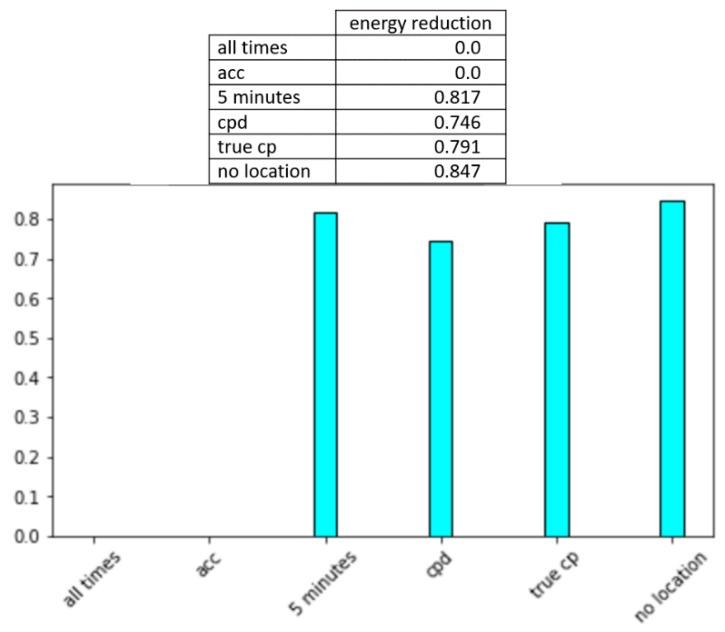
Percentage reduction in energy consumption in comparison with continuous sampling of movement and location.

From these consumption numbers, we can estimate the percentage decrease in energy consumption that is offered by all strategies, using continual location samplings as a baseline. Figure 12 plots these values. The calculations assume that the watch performs normal operations continuously, movement is sampled at 100 Hz, and locations are sampled as directed by the corresponding method. By comparing Figure 11 and Figure 12, we see the intuitive relationship between increased sampling, increased recognition accuracy, and increased energy consumption.

**Figure 13:**
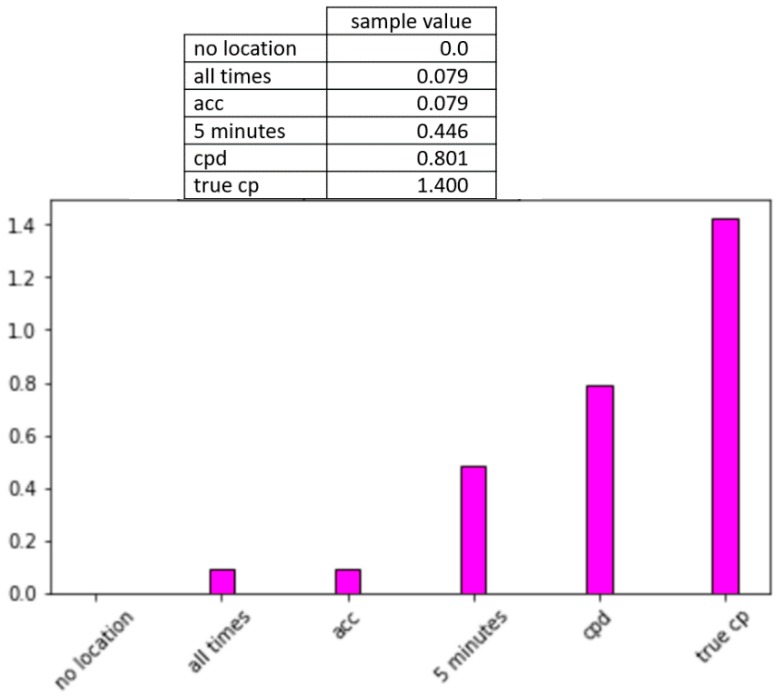
Comparison of value per sample between the no location, continuous location, acceleration-based location, every 5 min location, change point location (CPAM), and true change point location sampling strategies.

We want to determine which of the methods provides the most activity model value per unit of energy consumption. For this, we calculate the ratio between percentage increase in f-score recognition performance and increase in percentage increase in energy consumption, using no location samples as a baseline. Figure 13 plots these results. This graph provides an indication of the value of the increased location samples in comparison with not using any location information. Thus, while all location sampling increases the energy footprint of the app, strategic selection of location values ensures that the extra energy consumption is most advantageous for a robust activity model. The figure also shows that while CPAM’s use of SEP change point detection is effective, there is room to further improve change point detection for wearable data and offer even greater value when sampling location.

**Figure 14:**
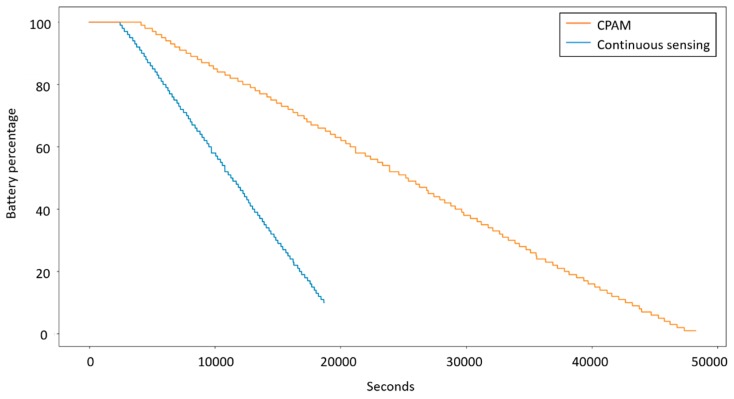
Average battery consumption using continuous and CPAM-based sampling of movement and location.

To obtain a practical perspective on energy consumption using CPAM for continual data collection, one subject wore a smartwatch continually for two weeks. The watch was charged each night and worn during the day until the battery was completed drained. Data was collected for one week with continuous sampling of movement data and location data, together with activity labeling. Data was collected for a second week using CPAM-based data sampling. Figure 14 graphs the averaged battery consumption using the two methods. Using continuous sensing, the battery drains in approximately 5 h. Using CPAM, data can be collected and labeled for almost 15 h without needing to charge the smartwatch.

### 4.6. Location Estimation from Activity Information

The experiments in this paper analyze the impact of change point detect-based location sampling on activity recognition performance and on battery consumption. In this final experiment, we turn the analysis around and use activity information to estimate location data. While we have focused on activity recognition as an application that can benefit from CPD, we posit that other location-based applications can improve their information reliability using activity recognition. When we downsample location, we assume that the location remains constant between samples. This assumption can lead to application errors. On the other hand, increasing the sample rate may drain the battery too quickly.

**Figure 15:**
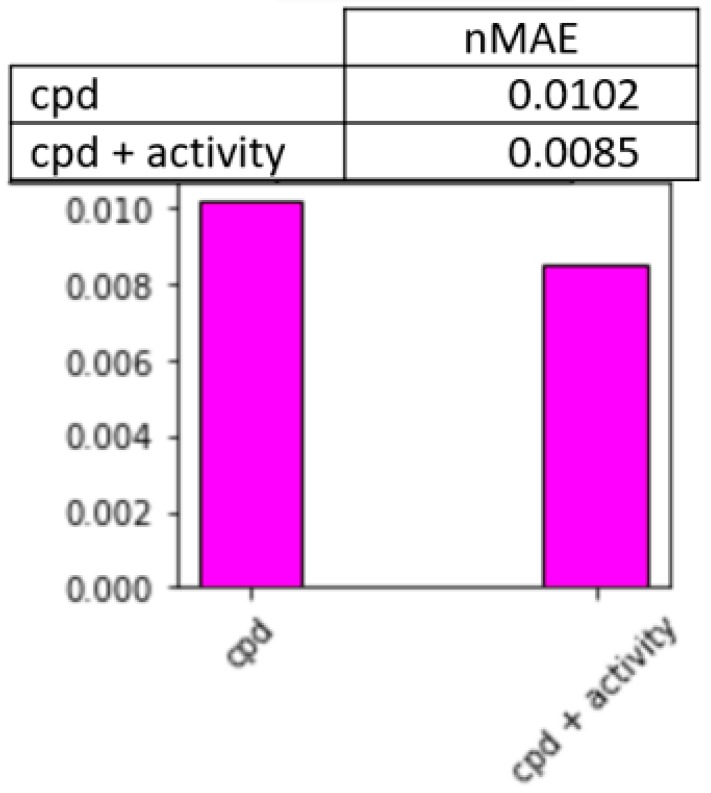
Normalized mean absolute error of location accuracy estimation between change points with and without activity information.

In this final experiment, we analyze the accuracy of location estimation using activity recognition. Specifically, we combine the location information sampled at a previous change point with the activity information (activity label and related activity features) to estimate location values between the sampled times. We compare this with location estimation that assumes the location remains constant between change points. Figure 15 plots the performance of these two location estimation approaches using normalized mean absolute error.

In this experiment, we did not utilize the ground truth activity labels provided by subjects. Instead, we used the activity labels that we created by the learned activity models. We did this to demonstrate an interesting synergy that exists between the learned concepts. Namely, smartwatch data can be input to a supervised learner to predict the activity class. At the same time, the predicted activity can be used to estimate location information for the remainder of the activity occurrence. This type of joint inference could strengthen predictions of other types of contextual information as well that are used by mobile applications.

## 5. Conclusions

In this paper, we introduce CPAM, an algorithm that detects change points in wearable sensor data to control data sampling rates. By strategically finding transitions between activity states, we support our hypothesis that change point-based sampling can support recognition of complex activities in real time while simultaneously reducing energy consumption. This work is vital because of the role that continual activity monitoring plays in health assessment and intervention as well as the design of activity-aware services.

Because location sampling is a large consumer of smartwatch battery resources, we focused on controlling location sampling in this paper. In future work, we can extend CPAM to control sampling rates for all of the collected information based on detected change points. The approach could potentially be further improved by predicting the duration of a detected activity and increasing sample rates when the end of an activity is near. We would also like to further explore the use of joint inference to improve the performance of related learning tasks including recognition of related activities, forecasting of activities and estimation of smartwatch and user state based on inferred activity contexts.

## Figures and Tables

**Table 1:** Activity categories.

Activity	Interpretation
Sleep	nighttime sleep (going to bed, waking up, nighttime interruptions), daytime naps
Work	work at office, work on computer, teach, attend class, finances, research, meetings
Eat	cook, eat at home, eat out, snack, drink, clean dishes
Errands	shop, doctor appointment, other appointment
Exercise	exercise machines, run, walk, bike, lift weights, sports
Travel	drive/ride in car, bus, train, airplane
Hygiene	dress, brush teeth, wash, bathe/shower, groom
Hobby	garden, games, care for others, care for house, socialize, entertainment, read

**Table 2:** Features extracted from smartwatch sensors.

**Sensor Data**	
Acc = <x acceleration, y acceleration, z acceleration>, rot = <yaw, pitch, roll>, course, speed, orientation, loc = <latitude, longitude, altitude>, heart rate, compass, date, time	
**Features**	**Data**
*f_statistical_*: max, min, sum, mean, standard deviation, mean absolute deviation, median absolute deviation, variance, zero crossings, interquartile range, coefficient of variation, skewness, kurtosis, entropy, discrete Fourier transform, signal energy, log signal energy, power, autocorrelation	acc, rot, course, speed, compass, heart rate
*f_relational_*: total, multidimensional correlation	acc, rot, loc
*f_temporal_*: day of week, hours, minutes, seconds past midnight	date, time
*f_navigational_*: heading change rate, stop rate, overall trajectory, distance travelled	loc, calculated for each window
*f_personal_*: frequent cluster membership, frequency/time cluster membership, distance from center	loc, calculated for each user
*f_positional_*: loc_type = <home, restaurant, road, store, work, attraction, service, other>	loc, calculated via reverse geocoding
**Activities**	
*A*: eat, errands, exercise, hobby, hygiene, sleep, travel, work, other	

**Table 3:** Data sample size for each activity category.

Activity	Number of Sensor Readings	Number of Occurrences
Eat	72,272	5253
Errands	6475	297
Exercise	48,984	5909
Hobby	29,400	8219
Hygiene	10,832	1455
Sleep	254,939	1038
Travel	25,022	3400
Work	224,012	14,518
Other	31,626	6141
**Total**	**703,284**	**46,230**

**Table 4:** SEP performance on smartwatch data collected for 66 subjects.

**SEP**	
True Positive Rate = 0.875	False Positive Rate = 0.150
G-Mean = 0.862	
**Baseline**	
True Positive Rate = 0.003	False Positive Rate = 0.46
G-Mean = 0.002	

**Table 5:** Energy consumption per second by normal watch operations, movement sampling, and location sampling.

Operation	Energy Consumption
Normal (1 s)	1.1430 × 10^−5^ Wh
Movement (1 sample)	1.3716 × 10^−5^ Wh
Location sample (1 sample)	7.6454 × 10^−5^ Wh
